# High Eg5 expression predicts poor prognosis in breast cancer

**DOI:** 10.18632/oncotarget.19215

**Published:** 2017-07-10

**Authors:** Qin Jin, Fang Huang, Xudong Wang, Huijun Zhu, Yun Xian, Jieying Li, Shu Zhang, Qichao Ni

**Affiliations:** ^1^ Department of Pathlogy, Affiliated Hospital of Nantong University, Nantong 226001, Jiangsu, China; ^2^ Surgical Comprehensive Laboratory, Affiliated Hospital of Nantong University, Nantong 226001, Jiangsu, China; ^3^ Health Insurance Office, Nantong University, Nantong 226001, Jiangsu, China; ^4^ Department of General Surgery, Affiliated Hospital of Nantong University, Nantong 226001, Jiangsu, China

**Keywords:** Eg5, BC, prognosis

## Abstract

Eg5 is a motor protein belonging to the kinesin-5 family and has been suggested to exert important function in tumors. In this study, we determined the mRNA and protein expression levels of Eg5 in cancerous and non-cancerous breast tissue by quantitative real-time polymerase chain reaction (qRT-PCR) and tissue microarray immunohistochemistry analysis (TMA-IHC) respectively. The results of 20 fresh-frozen BC samples demonstrated that Eg5 mRNA levels were significantly higher in BC tissues compared with corresponding non-cancerous tissue (*p* = 0.0009). TMA-IHC analysis in 127 BC tissues revealed that Eg5 expression obviously correlated with clinicopathologial parameters, including tumor grade (*p* = 0.004), ER status (*p* = 0.030), Ki67 status (*p* = 0.005), molecular classification (*p =* 0.026), N stage (*p* = 0.015), and TNM stage (*p* = 0.001). Kaplan-Meier survival curve indicated that high Eg5 expression (*p =* 0.012), Ki67 status (*p* = 0.014) and TNM stage (*p* = 0.026) were independent factors to predict poor prognosis for patients with breast cancer. Our data suggest that Eg5 is not only overexpressed in BC, it may be also served as a potential prognostic marker.

## INTRODUCTION

Breast cancer (BC) is one of the most common types of cancers among women; it affects over 1.3 million new patients and causes nearly 0.5 million deaths each year [1]. In China, the incidence of BC has increased approximately 30%, and the BC related mortality has doubled over the past 30 years [2–3]. BC is usually classified into several types on the basis of molecular signatures, clinicopathological characteristics, and treatment responses [4]. Despite remarkable advances in the diagnosis and therapy, the outcome of BC remains unsatisfactory [5–6]. A large number of BC criteria are routinely and widely acknowledged in the clinical field, including estrogen receptor (ER), progesterone receptor (PR), human epidermal growth factor receptor 2 (Her2), Ki67 proliferative index, TNM stage, tumor grade, metastatic status and molecular classification have been suggested for prognosis [7]. Thus, novel biomarkers are needed to optimize the treatment strategy, evaluate the therapeutic effectiveness, and predict the clinical outcomes of BC.

Eg5, also known as kinesin-5, KSP, or KIF11, is a microtubule-dependent motor protein encoded by KIF11 gene located on human chromosome 10. It plays an essential role in bipolar spindle formation and maintenance in early prometaphase [8–9]. Eg5 is composed of three domains: a motor domain at the N-terminus, a coil stalk domain in the middle, and a tail domain at the C-terminus, and the N-terminus motor domain works with the C-terminus tail domain to form a homotetrameric structure [10]. Correct mitosis is the basis of cell proliferation. The correct formation of spindle is the premise for mitosis. Eg5 regulates spindle formation mainly by the following two mechanisms: (1) KSP reverse the alignment of the anti parallel array of microtubules; (2) by connecting two microtubules in the same direction and moving to their ends, KSP binds with microtubule, which promotes spindle formation [11]. MEg5 of the central microtubules move transient away to the spindle poles. On the contrary, The point-like mEg5 of the astral microtubules moves transient to the microtubule negative in the early stage of mitosis. But in the later period, it moves to the positive electrode. This is the main reason for the polarity accumulation and redistribution of Eg5 in the early stage of mitosis. So that the understanding the role of Eg5 in mitosis [12]. Usually, Eg5 expression in CD34^+^ cell, testis, cardiac muscle cell and some other human normal proliferation tissues. However, the expression level was significantly lower than that in malignant tissues. Studies have evaluated the Eg5 expression and its correlation with clinicopathological characteristics in various malignant tumors. For example, activation of Eg5 expression contributes to the onste of B-cell leukemia [13], Eg5 over-expression has been reported in solid tumors such as lung cancer [14], renal cell carcinoma [15], Metastatic castrate-resistant prostate cancer(mCRPC) [16], non-muscle invasive bladder urothelial carcinoma [17], laryngeal squamous cell carcinoma [18], high grade astrocytic neoplasm [19]; in addition, five human pancreatic cancer cell lines (PANC1, EPP85, BxPC3, CFPAC1 and AsPAC1) [20] and ER-positive human breast cancer MCF-7 cell line [21] were also observed higher Eg5 expression, and the inhibition of Eg5 expression blocks cell cycle and inhibits cell proliferation [22–23]. Moreover, transgenic mice over-expressing Eg5 exhibit a high tendency to develop several types of malignancies [24]. These results indicate that Eg5 has a significant carcinogenic effect; in addition, Eg5 may be used as a novel biomarker of cancer therapy. Nevertheless, the relationship between Eg5 expression and the clinicopathological significance of BC has not been investigated.

In this retrospective study, our goal was to determine Eg5 expression between BC tissues and corresponding non-cancerous tissues, and to determine whether Eg5 could be used as a prognostic marker for BC patients. The mRNA level of Eg5 was detected using quantitative real-time polymerase chain reaction (qRT-PCR) analysis while the protein expression of Eg5 was measured by immunohistochemistry (IHC) analysis. In addition, the relationship between Eg5 expression and significant clinicopathological parameters of BC was evaluated.

## RESULTS

### Eg5 mRNA expression in BC patients by qRT-PCR test

**Figure 1:**
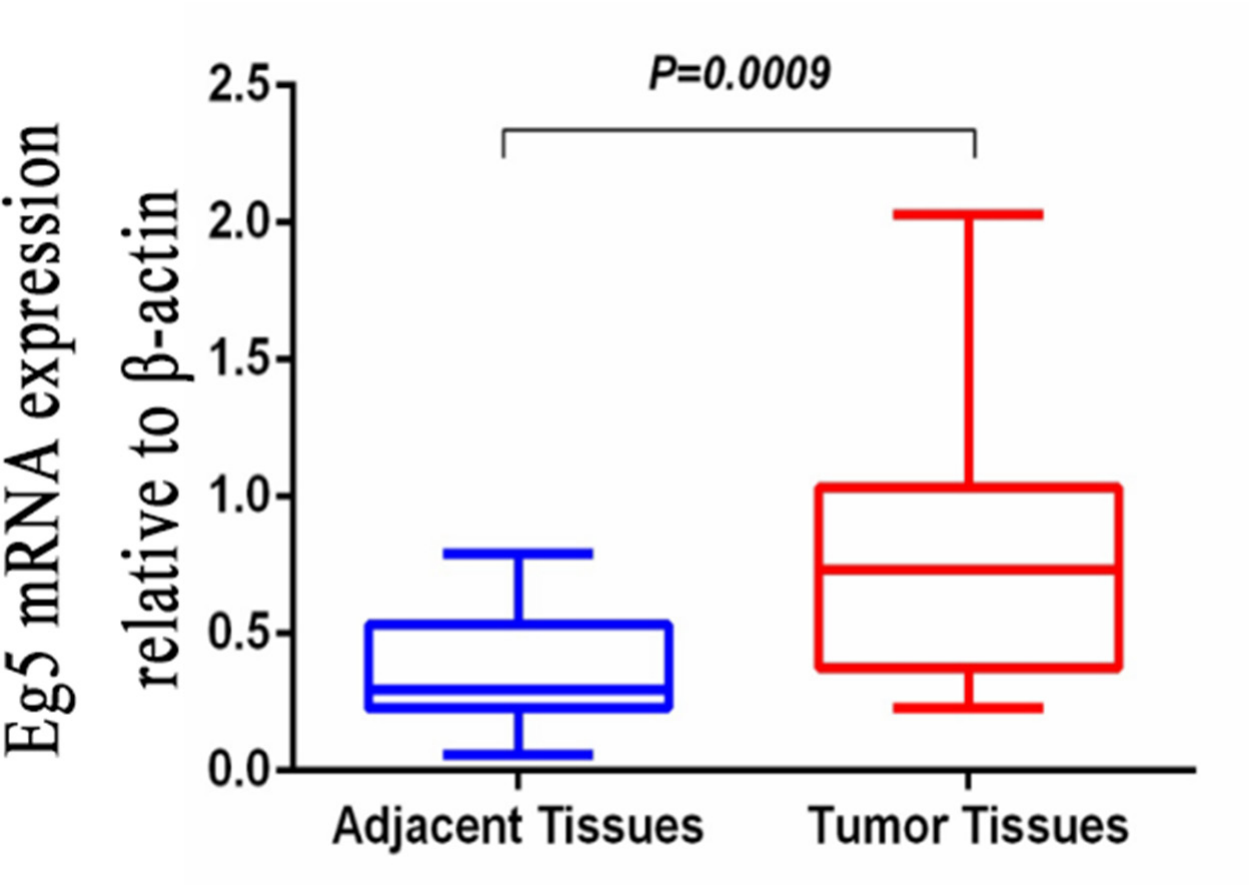
Quantitative real-time polymerase chain reaction (qRT-PCR) was employed to detect Eg5 mRNA expression levels in BC tissues and compared with corresponding non-cancerous tissues When normalized to β-actin mRNA levels, the Eg5 mRNA level in BC tissue (0.8145 ± 0.1153) is significantly higher than that in corresponding non-cancerous tissue (0.3660 ± 0.0469).

The expression of Eg5 mRNA was analyzed by qRT-PCR in BC tissue samples as well as in corresponding non-cancerous tissue samples obtained from 20 BC patients. Eg5 transcript levels were significantly higher in BC tissues compared with corresponding non-cancerous tissues (0.8145 ± 0.1153 *vs* 0.3660 ± 0.0469, 2.23-fold, t = 3.603, *p* = 0.0009. Wilcoxon signed-rank nonparametric test) (Figure 1).

### Expression of Eg5 protein in BC by IHC test

**Figure 2:**
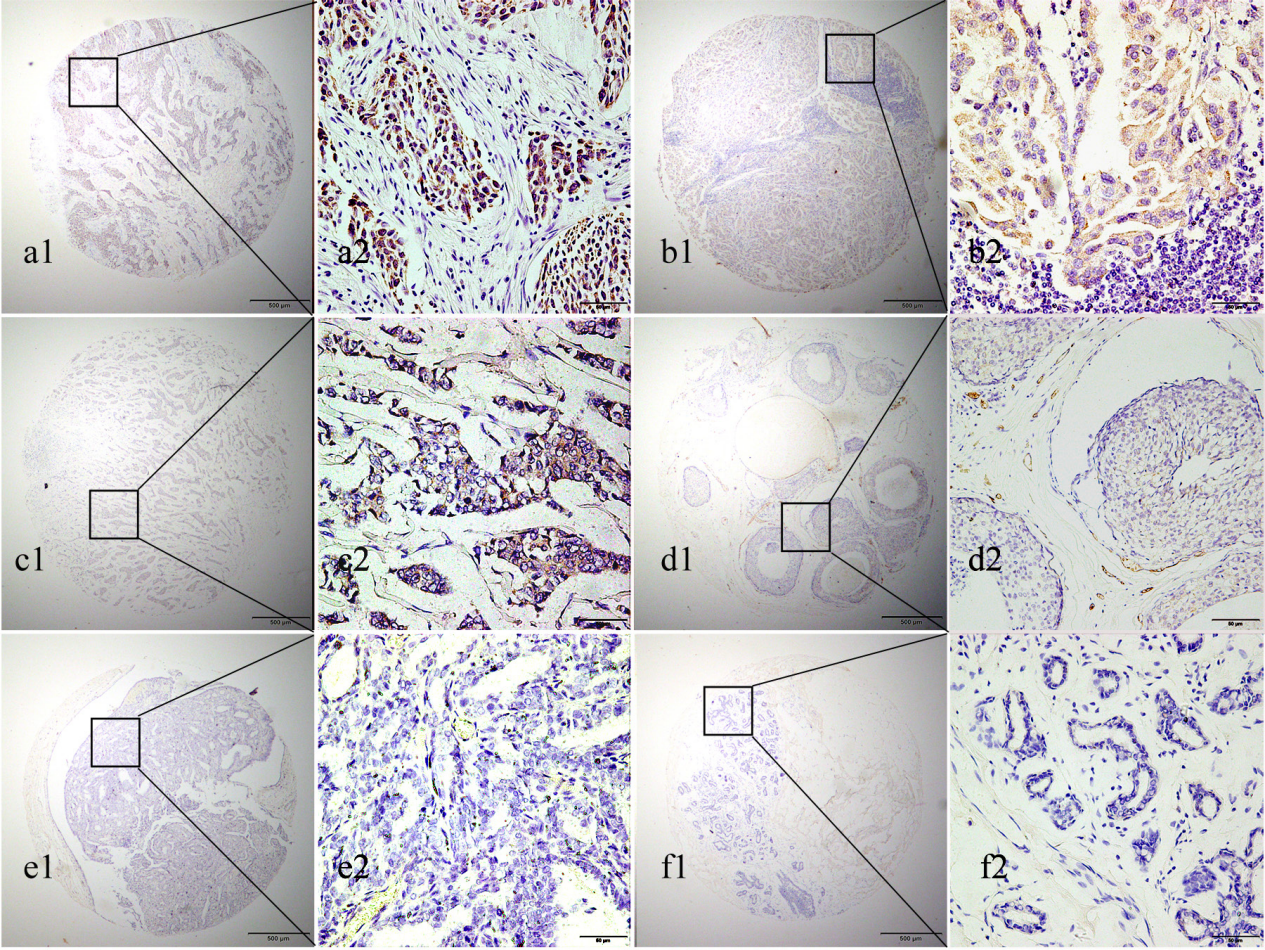
Representative images of Eg5 protein expression in BC and corresponding non-cancerous tissues with tissue microarray (TMA) **(a1, b1, a2 and b2)** High IHC staining of Eg5 in the cytoplasm of invasive breast cancer cells. **(c1 and c2)** Low IHC staining of Eg5 in the cytoplasm of invasive breast cancer cells. **(d1 and d2)** No IHC staining of Eg5 in the invasive ductal breast cancer cells. **(e1 and e2)** No IHC staining of Eg5 in the breast ductal papilloma cells. **(f1 and f2)** No IHC staining of Eg5 in the breast adenosis cells. Original magnification ×40 in (a1, b1, c1, d1, e1, f1); ×400 in (a2, b2, c2, d2, e2, f2).

To determin the protein expression of Eg5, TMA- immunohistochemistry analysis was performed. As shown in Figure 2, Eg5 was detected primarily in the cytoplasm of BC cells. High Eg5 expression was detected in 57.5% (73/127) of BC samples, significantly higher(χ2= 28.722, *p* < 0.001) than in 24.4% (31/127) of non-cancerous samples.

### Relationship between Eg5 protein expression and clinicopathological attributes of BC

Subsequently, the relationship between Eg5 protein levels and clinicopathological attributes of BC patients was investigated (Table 1). High expression of Eg5 was significantly associated with tumor grade (*p* = 0.004), ER status (*p* = 0.030), Ki67 status (*p* = 0.005), molecular classification (*p* = 0.026), N stage (*p* = 0.015) and TNM stage (*p* = 0.001). However, Eg5 protein expression was not significantly associate with age, tumor size, PR status, and Her2 status (Table 1).

**Table 1:** Association of Eg5 expression with clinical characteristics and selected biological markers of BC

Characteristic	n	Eg5 expression(%)		*Χ*^*2*^	P
		Low or no	High		
**Age (years)**				0.688	0.749
≤40	9	4 (44.4)	5 (55.6)		
40-60	76	30 (39.5)	46 (60.5)		
≥60	42	20 (47.6)	22 (52.4)		
**Tumor size (cm)**				1.760	0.185
≤2cm	57	21 (36.8)	36 (63.2)		
>2cm	70	34 (48.6)	36 (51.4)		
**Tumor grade**				8.345	0.004*
I-II	78	41 (52.6)	37 (47.4)		
III	49	13(26. 5)	36 (73.5)		
**ER**				4.701	0.030*
Negative	43	24 (55.8)	19 (44.2)		
Positive	84	30 (35.7)	54 (64.3)		
**PR**				0.747	0.387
Negative	72	33 (45.8)	39 (54.2)		
Positive	55	21 (38.2)	34 (61.8)		
**Her2**				0.181	0.670
Negative	82	36 (43.9)	46 (56.1)		
Positive	45	18 (40.0)	27 (60.0)		
**Ki67**				7.850	0.005*
Low	57	32 (56.1)	25 (43.9)		
High	70	22 (31.4)	48 (68.6)		
**Molecular classification**				9.280	0.026*
Luminal A	46	20 (43.5)	26 (56.5)		
Luminal B	38	10 (26.3)	28 (73.7)		
Her2-overexpression	29	14 (48.3)	15(51.7)		
TNBC	14	10 (71.4)	4 (28.6)		
**N stage**				5.952	0.015*
N0	48	27 (56.2)	21 (43.8)		
N1+2+3	79	27 (34.2)	52 (65.8)		
**TNM stage**				11.153	0.001*
Stage I-II	88	46 (52.3)	42 (47.7)		
Stage III	39	8 (20.5)	31 (79.5)		

*p < 0.05

### High Eg5 protein expression predict poor prognosis of BC patients

**Figure 3:**
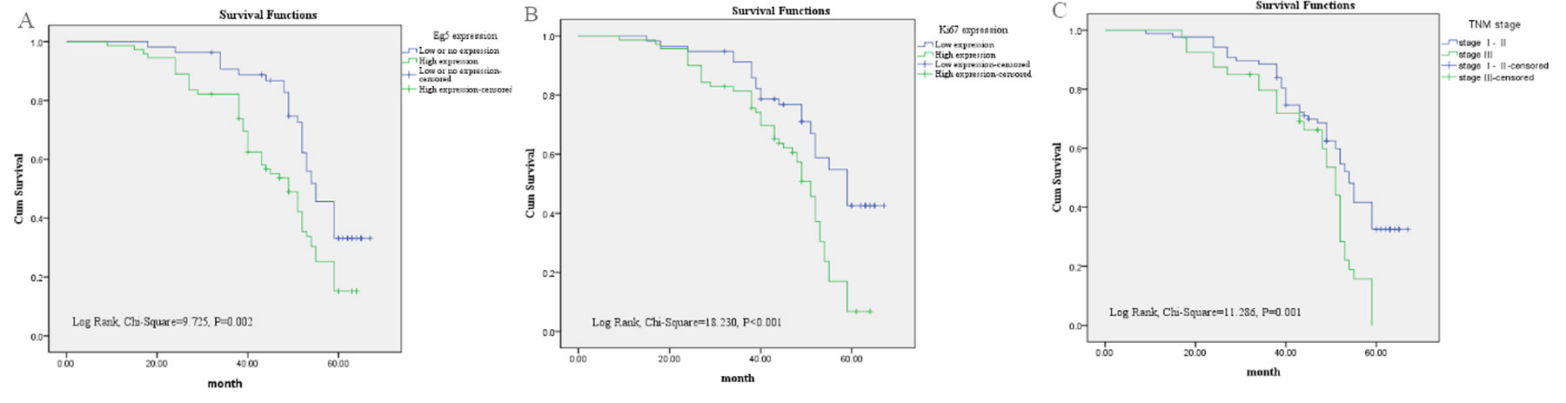
Survival analysis of BC patients by Kaplan-Meier method **(A)** Overall survival rate in BC patients with high cytoplasmic expression of Eg5 (green line) was statistically lower than that in BC patients with low and no Eg5 expression (blue line). **(B)** Overall survival rate in BC patients with high Ki67 expression (green line) was statistically lower than that in BC patients with low Ki67 expression (blue line). **(C)** Overall survival rate in BC patients with advanced TNM stage III (green line) was statistically lower than that in BC patients with early TNM stage I-II (blue line).

Univariate analysis was used to evaluated Eg5 protein expression and other clinicopathologic factors on prognosis of BC. High level of Eg5 protein expression (HR 1.908; *p* = 0.003), Her2 status (HR 1.705; *p* = 0.014), Ki67 statue (HR 2.473; *p* < 0.001) and TNM stage (HR 2.306; *p* = 0.001) were significant associated with poor overall survival (Table 2). These prognostic factors were further evaluated by multivariate Cox proportional hazards regression model analysis. High Eg5 expression (HR 1.724 *p* = 0.012), Ki67 status (HR 1.837; *p* = 0.014) and TNM stage (HR 1.676; *p* = 0.026) were all independent prognostic markers of poor 5-year overall survival (Table 2). Using the Kaplan-Meier analysis, which is used to assess the survival of BC patients. BC patients with high expression of Eg5 protein had significantly shorter overall survival (P = 0.002) compared with those with low or no Eg5 expression (Figure 3A), and patients with a high Ki67 expression had a poorer overall survival (P < 0.001) than patients with Ki67-low tumors (Figure 3B), patients in TNM stage III had a lower overall survival (P = 0.001) than patients in TNM stageI-II (Figure 3C).

**Table 2:** Univariate and multivariate analysis of prognostic factors in BC for 5-year overall survival

	Years	Univariate analysis			Multivariate analysis		
		HR	*p*	95% CI	HR	*p*	95% CI
**Expression of Eg5**							
High vs low or no expression	5	1.908	0.003*	1.238-2.943	1.724	0.012*	1.028-2.635
**Age (years)**							
≤40 vs 40-60 versus ≥60	5	1.194	0.342	0.828-1.722			
**Tumor grade**							
I- II vs III	5	1.208	0.378	0.793-1.840			
**Tumor size (cm)**							
≤2cm vs>2cm	5	1.093	0.675	0.720-1.661			
**Expression of ER**							
Negative vs Positive	5	0.768	0.225	0.504-1.171			
**Expression of PR**							
Negative vs Positive	5	0.696	0.104	0.450-1.078			
**Expression of Her2**							
Negative vs Positive	5	1.705	0.014*	1.116-2.605			
**Expression of ki67**							
Low vs High	5	2.473	<0.001*	1.580-3.869	1.837	0.014*	1.128-2.991
**Molecular classification**							
Luminal A vs Luminal B vs Her2-overexpression vs TNBC	5	1.184	0.073	0.985-1.423			
**N stage**							
N0 vs N1+2+3	5	1.048	0.831	0.684-1.604			
**TNM Stage**							
Stage I- II vs Stage III	5	2.036	0.001*	1.502-3.539	1.676	0.026*	1.063-2.642

*p < 0.05

## DISCUSSION

We know Eg5 mainly related to chromosome localization, centrosome separation and the formation and separation of bipolar spindle. The increasing high expression of Eg5 disturbs the normal assembly of the spindle and the balance of power associated with its function, which eventually leads to the loss of spindle, genomic instability and tumor. In this study, the expression of Eg5 was widely found in BC, and the results were consistent with the experimental results of Eg5 expression in several other malignant tumors. Eg5 has been implicated in tumorigenesis [25–26] because it is overexpressed and activated in leukemia; Eg5 also triggers genomic instability in transgenic mice. Hayashi et al. reported that Eg5 expression levels were proportional to the mitotic population in prostate cell lines [22]; monitoring of cells in untreated and HR22C16-A1 treated MCF-7 by using live cell confocal imaging techniques GFP-tubulin to compare the difference between the formation of microtubule spindle and cell division. Marcus et al. fund that the untreated cells, microtubules formed a bipolar spindle, then mitosis is shown after cytokinesis. The cells after HR22C16-A1 treatment, which has been found that bipolar spindle could not be formed. They indicated that Eg5 expression levels were positively associated with the mitotic index of BC [27]; Tang et al. also showed that Eg5 expression could be detected in all cases of head and neck squamous cell carcinoma, and the Eg5 expression level is correlated with cancer cell proliferative activity [28]. Consistent with previous studies, our study revealed that the Eg5 expression is correlated with malignant behaviors of BC development. Researchers using RNAi interference technology, Eg5 antibody neutralization and small molecule inhibitors of Eg5 can block tumor cells in metaphase of mitosis, and then lead to apoptosis. Several Eg5 inhibitors decrease cancer growth and cause tumor regression [29–31], such as Eg5 small molecule inhibitors can prevent pancreatic cancer cells from mitosis to stop pancreatic cancer cells and induce apoptosis. The molecular mechanism of Eg5 in tumorigenesis has been investigated; it is modulated by Parkin via the Hsp70-JNK-c-Jun signaling pathway [10]; the Eg5 mutation can induce drug resistance in cancer cell lines [32]; Eg5 inhibitor causes mitotic arrest in tumor cells through activation of c-Jun NH2 kinase pathway [33]. These data suggest that Eg5 represents an attractive drug target for cancer treatments [34–35]. Our study aimed to determine whether Eg5 plays a similar oncogenic role in BC and whether Eg5 can be used as a new biomarker for patients with BC.

In this study, we compared the expression of Eg5 in BC tissue samples with matched non-cancerous samples. We observed that both Eg5 mRNA and protein levels were significantly higher in BC tumor samples than in the corresponding adjacent non-cancerous tissues. TMA-IHC analysis revealed that Eg5 expression obviously correlated with clinicopathologial parameters, including tumor grade, ER status, Ki67 status, molecular classification, N stage and TNM stage. Eg5 protein was predominantly localized in the cytoplasm of BC cells and all these results are consistent with previous findings in other types of cancer [14, 28]. The results of Kaplan-Meier survival analysis show that patients with high Eg5 expression had lower survival. In addition, Cox regression analysis showed that Eg5 was an independent prognostic factor, and maybe played an important regulators involved in the development of BC.

To date, there are only a few studies reporting the prognostic significance of Eg5 in human cancers. In our study, high Eg5 protein was associated with poor overall survival for BC patients, which indicated that Eg5 protein level may be used as an independent marker in predicting unfavorable prognosis. Similar results were observed in two previous studies, which implied that a high Eg5 expression corresponds to a short survival time in bladder and prostate cancer patients [16–17].

Michel et al. reported a significant nuclear expression of Eg5 in prostate cancer [16]. In contrast, we mainly observed the cytoplasmic expression of Eg5 in BC tissues, and rarely detected the nuclear expression of Eg5 in BC tissues, These differences are probably due to the differences in tumor types and antibodies used. Future *in vitro* and *in vivo* studies that include larger number of samples are necessary to further investigate the role of Eg5 in BC tumor biology.

Taken together, we analyzed the Eg5 expression and demonstrated that Eg5 is involved in the BC development. The findings indicate that Eg5 is an independent prognostic factors of BC and Eg5 may be identified as a novel prognostic biomarker in BC patients. Relative studies concerning the potential mechanisms of Eg5 in BC and other kinds of human cancers are being performed by our research group.

## MATERIALS AND METHODS

### Patient specimens

20 fresh-frozen BC tissues and corresponding non-cancerous tissues were collected from the Department of Pathology, the Affiliated Hospital of Nantong University. Simultaneously, a total of 127 paraffin-embedded BC tissue samples and 127 matched non-cancerous tissue samples were collected from the Department of Pathology, the Affiliated Hospital of Nantong University, between January 2007 and January 2010 to construct the tissue microarray (TMA). Diagnosis of BC was confirmed according to the latest World Health Organization criteria [2].

All 127 patients underwent mastectomy and/or axillary dissection (radical or functional, based on clinical and surgical findings). Lymph node metastasis was confirmed by postoperative histological examination. The patients’ clinical information were collected simultaneously, including patient age, tumor grade, tumor size, hormone receptor status, Her2 expression status, Ki67 status, N stage, and TNM stage. In regards to molecular classification, due to the fact that chips of genotyping are rather expensive, the majority of the professionals in Inernational Conference of Breast Cancers in St. Gallen in 2011 came to the conclusion that the chips pattern of genotyping could be replaced by chemical staining results of immune cells according to the ER, PR, Her2, and Ki67. According to the 2015 ST Gallen International Breast Cancer Conference [36], the cases were classified into four subtypes: Luminal (Luminal A and Luminal B), Her2-overexpression, basal-like and normal-breast-like. Triple negative breast cancer (TNBC) is the expression of BC when ER, PR, Her2 are all negative. But there are differences on gene expression profile and immune phenotype between TNBC and basal-like group. These two can not be exactly the same. Meanwhile, TNBC also includes part normal bresat group. Pathological stage was based on the seventh edition of the American Joint Committee on Cancer Classification (AJCC). Samples of Ki67 expression less than 14% were considered Ki67 negative [37]. None of the patients received radiotherapy or chemotherapy before surgery. Each patient signed written informed consent for this present study. The study protocol was approved by the Ethics Committee of the Affiliated Hospital of Nantong University and all experiments were performed in accordance with approved guidelines of the Affiliated Hospital of Nantong University.

### Quantitative real-time polymerase chain reaction (qRT-PCR)

20 fresh BC tissue samples and corresponding non-cancerous tissue samples were used for qRT-PCR test as previously described [38]. Total RNAs were extracted using Trizol reagent (Invitrogen, Carlsbad, CA) following the manufacturer's protocols. qRT-PCR was performed using SyberGreen on an ABI 7500 thermal cycler (Applied Biosystems). The PCR procedures were as follows: UDG pre-treatment at 50 °C for 2 min, 1 cycle; initial denaturation at 95 °C for 10 min; denaturation at 95 °C for 15s, annealing and extension at 60 °C for 60s, 40 cycles. All experiments were performed in triplicate. The primers for Eg5 were as follows: forward primer 5′-GAA CAA TCA TTA GCA GCA GAA-3′ and reverse primer 5′-TCA GTA TAG ACA CCA CAG TTG-3′. The β-actin was used as an internal control, and the primers for β-actin were as follows: forward 5’-TAA TCT TCG CCT TAA TAC TT-3’, reverse 5’-AGC CTT CAT ACA TCT CAA-3’. The relative Eg5 mRNA expression was calculated using the 2^-ΔΔCt^ method.

### Immunohistochemistry (IHC) analysis

IHC analysis was performed as described previously [39]. Antigen retrieval was achieved by boiling under pressure in citrate buffer (pH 6.0). Non-specific binding was blocked through incubation with 5% goat serum in PBS for 15 min. TMA sections were incubated with a polyclonal anti-Eg5 antibody (1:100, abcam, Cambridge, USA) and subsequently with Envision goat anti-rabbit HRP secondary antibody (DAKO, Carpinteria, CA). The IHC analysis was performed at the same time under the same conditions. Immunostained sections were scored by two independent pathologists under blinded experimental conditions according to intensity and percentage of Eg5-positive cells.

The intensity of Eg5-positive cells was scored as follows:0, 1, 2, or 3, from negative, weak, moderate, and strong intensity. The percentage of Eg5-positive cells was scored as follows: 0 for no cytoplasm expression, 1 for 1-25% positive tumor cytoplasm, 2 for 26-50% positive tumor cytoplasm, 3 for 51-75% positive tumor cytoplasm, and 4 for 76-100% positive tumor cytoplasm. The cutoff point for a statistically significant Eg5 expression score in terms of OS was set using the X-tile software program (The Rimm Lab at Yale University; http://www.tissuearray.org/rimmlab) [40]. The multiply of the intensity and percentage scores led to as the final Eg5 staining score and was defined as follows: staining score less than 6 considered as low expression, while staining score of 7 or more was considered as high expression.

### Statistical analysis

The mRNA expression of Eg5 in fresh BC tissues and corresponding non-cancerous tissues was calculated by the Wilcoxon signed-rank nonparametric test. Pearson’s *χ*2 was conducted to examine the correlation between Eg5 protein expression and clinicopathological parameters. Kaplan-Meier and log-rank test were performed to calculate the survival curves. Factors that of prognostic significance in the univariate analysis were further analyzed using a multivariate Cox regression model. For all tests, *p*-values less than 0.05 were considered statistically significant. All statistical works were analyzed using STATA Version 12.0 software (Stata Corporation, College Station, TX).
